# Intravenous versus intracuff alkalinized lidocaine to prevent postoperative sore throat: a prospective randomized controlled trial

**DOI:** 10.11604/pamj.2024.48.18.40317

**Published:** 2024-05-24

**Authors:** Salma Ketata, Yassine Maktouf, Imen Zouche, Sarhane Feki, Mariem Keskes, Ayman Trigui, Amira Akrout, Rahma Daoud, Amine Chaabouni, Hichem Cheikhrouhou

**Affiliations:** 1 Department of Anesthesiology, Habib Bourguiba University Hospital, Sfax, Tunisia,; 2 Department of Abdominal Surgery, Habib Bourguiba University Hospital, Sfax, Tunisia

**Keywords:** Intravenous lidocaine, intracuff lidocaine, general anesthesia, cough, endotracheal intubation, postoperative sore throat

## Abstract

**Introduction:**

postoperative sore throat (POST) is a common complication after general anesthesia with endotracheal intubation caused by tracheal mucosal injury. Multiple techniques prevent postoperative sore throat (POST). Our study aimed to compare two techniques: intravenous and intracuff lidocaine versus placebo to prevent postoperative sore throat after general anesthesia with orotracheal intubation.

**Methods:**

we conducted a prospective double-blind, randomized controlled clinical trial involving patients, proposed for a scheduled surgery less than 240 minutes under general anesthesia with orotracheal intubation. Patients were divided into three groups: L group: infused with saline, cuff filled with alkalinized lidocaine. S group: Infused with 1.5 mg/kg of lidocaine, cuff filled with saline. T group: placebo: infused with saline, cuff filled with saline. Our primary outcome was the incidence of sore throat and their (visual analog scale) VAS score in the first 24 postoperative hours. Our secondary outcomes were the incidence of cough, dysphonia, dysphagia, and postoperative nausea and vomiting.

**Results:**

ninety patients were analyzed and divided into 3 groups of 30. The incidence of POST at the sixth postoperative hour, for placebo, the “L” group, and the “S” group, respectively, was 67%, 30%, and 47%. And at the 24^th^ postoperative hours 67%, 13%, and 37%. Intravenous lidocaine reduced significantly the VAS of POST at the 24^th^ hour (S: 6.80 ± 20.70; T: 20.67 ± 18.182; p= 0.02). Alkalinized lidocaine decreased significantly the VAS of POST in the sixth (L: 8.17 ± 22.761; T: 23 ± 21.838; p = 0.048) and the 24^th^ postoperative hour (L: 6.33 ± 20.592; T: 20.67 ± 18.182; p= 0.019) with the lowest pain score. There was no statistically significant difference between the L and S groups at the 6 and 24 postoperative hours. Both lidocaine techniques reduced cough at emergence, with the superiority of alkalinized lidocaine (p=0.02). They decreased the incidence of cough, dysphonia, dysphagia, nausea, and vomiting compared to a placebo.

**Conclusion:**

intravenous and intracuff lidocaine allowed better control of postoperative sore throat.

## Introduction

Postoperative sore throat (POST) is frequent after general anesthesia with endotracheal intubation (40%) [1]. A tracheal mucosal injury could be the cause. These injuries can influence the extubation process and may be responsible for complications such as excessive coughing or buckling on the tube, increasing cerebral pressure, intraocular pressure, intraabdominal and/or systemic blood pressure, myocardial ischemia, surgical bleeding, tachycardia, and bronchospasm [2]. Several methods are used to control posts such as cuff pressure control, systemic analgesics and antihyperalgesics, systemic or local instillation of corticosteroid sprays and gels [3], gargling of ketamine or anti-inflammatory drugs [4], and the use of local anesthetic solutions to inflate the endotracheal tube (ETT) cuff [2,5,6]. In this trial, our objective was to compare two different techniques using lidocaine (intravenous and intracuff) versus placebo to prevent POST.

## Methods

**Study design:** we conducted a prospective double-blind, randomized, and controlled clinical trial in the operating room of the anesthesia unit of Habib Bourguiba involving patients proposed for elective surgery under general anesthesia with orotracheal intubation.

**Patients selection:** we included patients over 18 years of age, with an American Society Of Anesthesiologists Physical Status Score (ASA) of 1, 2, or 3 proposed for elective surgeries under general anesthesia with orotracheal intubation (OTI), and surgery time less than 240 minutes. The non-inclusion criteria were patient refusal, predicted difficult airway management, upper airway surgery, recent airway inflammation, injury or tumor, contraindication of protocol drugs, and laparoscopic surgery. The exclusion criteria were Intubation time exceeding 240 minutes, difficult or traumatic management of the airway, accidental extubation, and modifications of the anesthetic protocol.

**Sample size:** the estimation of the sample size was carried out based on a pilot study and the publications of Estebe and Dollo [7-9]. We have estimated that the use of lidocaine would reduce the incidence of POST by 30% compared to a placebo. For a type I error of 0.05 and 80% power, we have estimated that each of the three groups must be formed by a minimum of 25 individuals. We chose to form three groups of 30 patients each.

**Study protocol:** for all groups, an anesthesiologist who did not participate in the study prepared a solution to be injected intravenously (syringe A=10ml of saline or 1.5mg/kg of lidocaine) and a solution to inflate the tube cuff (syringe B=10ml of saline or alkalinized lidocaine) according to randomization. In the operating room, patients were monitored by an electrocardioscope, pulse oximetry, and noninvasive blood pressure monitoring. A 20 gauge vein needle was placed and patients received 10 cc/kg of 0.9% isotonic saline. Preoxygenation was performed for 3 minutes with 100% oxygen. After injecting syringe A, induction was performed with fentanyl 3μg/kg, propofol 2,5mg/kg, and cisatracurium 0,15mg/kg. Intubation was performed after 3 min by a trained anesthesiologist using direct laryngoscopy. The women were intubated with 7mm tubes and the men with 7,5mm tubes. Endotracheal tube (ETT) cuffs were inflated with the liquid of syringe B with a minimal volume needed to stop the leak of air. Anesthesia was maintained with propofol 5mg/kg associated with fentanyl 1μg/kg and cisatracurium 0,05mg/kg every 30 minutes. The patients were mechanically ventilated with 50% oxygen and 50% air, and 6-8 ml/kg of tidal volumes to maintain the end-tidal CO2 concentration at 30-35 mm Hg. Thirty minutes before the end of the procedure, patients got 1g of paracetamol and 3mg of morphine. Extubation was performed in the operating room. The volume of the cuff removed was recorded. The patients were transferred to the post-anesthesia care unit for 2 hours. All patients were admitted to their original department for at least 24 hours. They received paracetamol 1g×4, nefopam 20mg×4, and tramadol 100mg×3. The follow-up of the patients was carried out by another anesthesiologist who didn´t know the enrollment groups.

**Data collection:** preoperative and intraoperative data was collected including demographics and comorbidities of the patients, ASA score, duration of surgery, and opioid doses. For postoperative data, we measured sore throat with a visual analog scale (VAS) at the 6^th^ and the 24^th^ postoperative hours. Cough was recorded at intubation, the emergence of anesthesia, and the 6^th^ then the 24^th^ postoperative hours. Other throat complaints such as dysphonia, dysphagia, and postoperative nausea and vomiting were evaluated with a binary scale (yes/no) and recorded at the 6^th^ and the 24^th^ postoperative hours.

**Outcomes:** our primary outcome was VAS pain in the throat during the first 24 postoperative hours. Secondary outcomes were the incidence of cough, dysphonia, dysphagia, and postoperative nausea and vomiting during the first 24 postoperative hours.

**Ethical considerations:** this study was conducted after approval of the Southern Protection Committee of People (C.P.P.SUD) under the aegis of the Health Ministry of the Tunisian Republic reference CPP SUD N°017/2019 and written informed consent of the patients.

**Statistical analysis:** the software IBM SPSS® 25.0.0.1 was used for statistical analysis. For quantitative variables that were compared between groups, after a means-based homogeneity of variance test, ANOVA was performed, followed by a Seffe post hoc test. The Kruskal-Wallis test was used for nonparametric data. Pearson´s chi-square test was used for the analysis of qualitative variables. Statistical significance was defined as p < 0.05.

## Results

**Figure 1:**
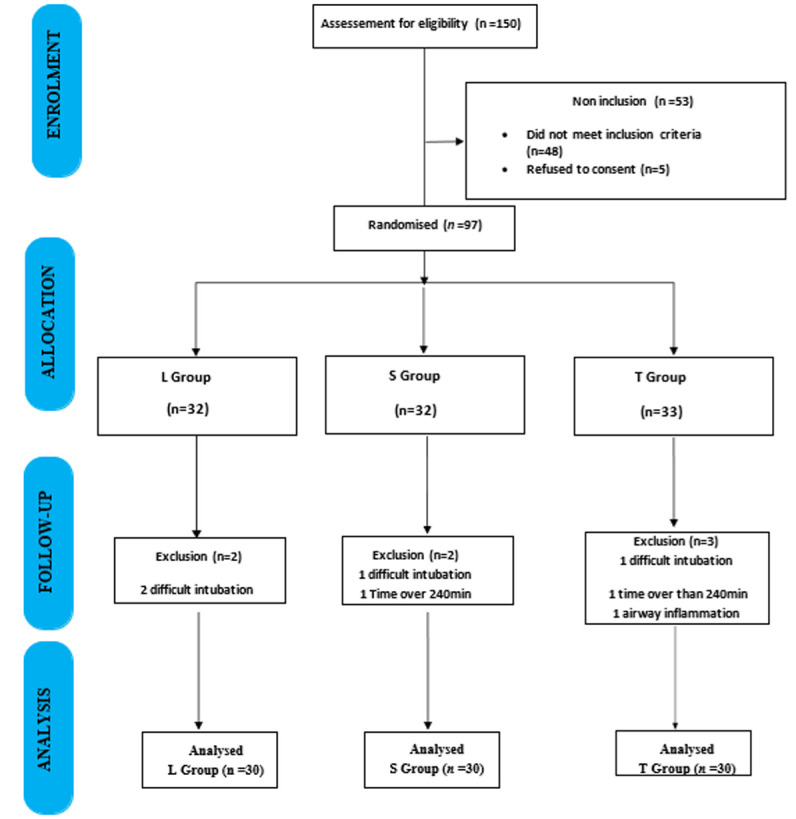
flow chart

Ninety-seven patients were included in this study. 7 patients were excluded: 4 for difficult intubation, 2 for surgery time greater than 240 min, and 1 for airway inflammation. We analyzed 90 patients divided into 3 groups of 30 (Figure 1).

**General characteristics:** our sample was characterized by a mean age = 50 ±16,59 years and a sex ratio(male/female) =1,36. There were no statistical differences between the three groups regarding the demographic parameters, comorbidities, surgery time, and opioid doses (Table 1). There was no difference in the initial volume injected into the cuff or in the volume removed. No cases of cuff rupture or signs of lidocaine toxicity have been reported.

**Table 1:** comparison of demographic data between the 3 groups

	T Group	L group	S group	P value
**Age (year) ±SD**	47.03±16.372	49.83±17.329	49,53±16,504	0.777*
**Weight (kg) ±SD**	74.87±14.33	74.83±14.788	74.07±12.654	0.969*
**Height (cm) ±SD**	166.733±9.3216	166.733±7.794	168.233±8.85	0.743*
**Sexe F/M (n)**	19/11	17/13	16/14	0.727 ¥
**ASA 1 (n)**	16	18	15	0.948 ¥
**ASA2 (n)**	11	9	12	
**ASA3 (n)**	3	3	3	
**HBP(+/-)**	5/25	7/23	7/23	0.475 ¥
**Diabetis(+/-)**	5/25	5/25	5/25	1.00 ¥
**COPD (+/-)**	4/26	5/25	6/24	0.787¥
**Smoking (+/-)**	7/23	7/23	4/26	0.304 ¥
**Surgery time (min)±SD**	127±25.3	123±26.42	124± 25.32	0.823*
**Fentanyl (µg/kg)±SD**	4.28±0.809	3.97±0.742	4.16±0.78	0123*

T Group: placebo, L Group: intracuff alkalinized lidocaine, S Group: intravenous lidocaine F: female, M: male, HBP: hight blood pressure, COPD: chronic obstructive pumonary disease, Kg: kilogram, cm: centimetre, min: minute, µg: microgram, (+/-): (present/absent), n: patient number, *: Anova test, ¥: Pearson’s chi-square test, SD: standard deviation

**Primary outcomes:** the incidence of POST at the sixth postoperative hour, for placebo, the “L” group, and the “S” group, respectively, was 67%, 30%, and 47%. And at the 24^th^ postoperative hours 67%, 13%, and 37%. VAS of the sore throat was significantly decreased for the L Group compared with the T Group at the 6th hour (L: 8.17 ± 22.761; T: 23 ± 21.838; p= 0.048) and at the 24^th^ hour (L: 6.33 ± 20.592; T: 20.67 ± 18.182; p= 0.019). However, it was significantly reduced only at the 24^th^ hour for the S group compared to the T group (S: 6.80 ± 20.70; T: 20.67 ± 18.182; p= 0.020). There was no statistically significant difference between the L and S groups at the 6 and 24 postoperative hours (Table 2).

**Table 2:** comparison of visual analogue scale (VAS) of sore throat between the 3 groups

	VAS	
	**H6**	**H24**
**Group T**	23 ± 21.838	20.67 ± 18.182
**Group L**	8.17 ± 22.761	6.33 ± 20.592
**Group S**	15.33 ± 24.174	6.80 ± 20.70
**P value**	P=0,048* p1 = 0.048 ƚp 2= 0.436 ƚ p3= 0,484#410;	P=0,015* p 1= 0.019 ƚ p2 = 0.020 ƚp3=0,724 ƚ

T Group: placebo, L Group: intracuff alkalinized lidocaine, S Group: intravenous lidocaine P: p value of 3 groups, P1: T Group versus L Group, P2: T Group versus S Group, P3: L Group versus S Group, *: Anova test, ƚ: scheffé post hoc test

**Secondary outcomes:** the incidence of cough at the time of intubation was 33%,17%, and 13% for the T group, the L group, and the S group respectively without significant difference between the 3 groups. It was 73%, 17%, and 43% respectively at the emergence of anesthesia. Compared to placebo, the intracuff alkalinized lidocaine and intravenous lidocaine significantly reduced the incidence of cough at the emergence of anesthesia (p<0,001 and p=0,018 respectively) with the superiority of the intravenous route (p=0,024) (Table 3). The incidence of cough at the 6th post-operative hour was 63%, 27%, and 20% for the T group, the L group, and the S group respectively. It was 53%, 13%, and 10% respectively in the 24^th^ postoperative hour. Compared to placebo, the incidence of cough in the 6th and 24^th^ postoperative hours decreased significantly when we used intracuff alkalinized lidocaine or intravenous lidocaine technique. There were no statistically significant differences between the L and S groups when we studied the incidence of postoperative cough (Table 3). Both the Intracuff alkalinized lidocaine and the intravenous lidocaine technique significantly reduced the incidence of dysphonia and dysphagia at the sixth and 24^th^ postoperative hours compared to placebo, but no differences were found between L and S group (Table 3). Both lidocaine techniques significantly reduced the incidence of nausea and vomiting in the 24^th^ postoperative hour compared to placebo. Only the intravenous bolus of lidocaine reduced them in the 6^th^ postoperative hour. No differences were found when comparing intravenous lidocaine with in-cuff alkalinized lidocaine (Table 3).

**Table 3:** comparison of the incidence of cough, dysphonia, dysphagia and postoperative nausea and vomiting between the 3 groups

Cough	T Group	L Group	S Group	P value
Intubation (+/-)	10/20	5/25	4/26	P = 0,126¥
Emergence of anesthesia (+/-)	22/8	5/25	13/17	P < 0,001¥ P1< 0,01¥ P2=0,018¥ P3=0,024¥
H6 (+/-)	19/11	8/22	6/24	P=0,001¥P1=0,004¥ P2=0,001¥ P3=0,542¥
H24 (+/-)				P < 0,001¥ P1=0,001¥ P2 < 0,001¥
**Dysphonia**	16/24	4/26	3/27	P3=0,688¥
H6 (+/-)	26/4	11/19	13/17	P < 0,001¥ P1< 0,001¥ P2< 0,001¥ P3=0,589¥
H24(+/-)		7/23	6/24	P< 0,001¥ P1< 0,001¥ P2< 0,001¥ P3=0,754¥
**Dysphagia**	26/4			
H6 (+/-)	21/9	10/20	10/20	P=0,004¥ P1=0,004¥ P2=0,004¥ P3=1¥
H24 (+/-)				P< 0,001¥P1< 0,001¥ P2< 0,001¥
**PONV**	22/8	6/24	6/24	P3=1¥
H6 (+/-)	19/11	14/16	8/22	P=0,017¥ P1=0,149¥ P2=0,004¥ P3=0,108¥
H24 (+/-)	17/13	8/22	7/23	P=0,012¥ P1=0,018¥ P2=0,008¥ P3=0,766¥

T Group: placebo, L Group: intracuff alkalinized lidocaine, S Group: intravenous lidocaine (+/-): (present/ absent), H6: 6^th^ postoperative hour, H24: 24th postoperative hour, PONV: postoperative nausea and vomiting, P: p value of the 3 groups, P1:T Group; versus L Group, P2: T Group versus S Group, P3: L Group versus S Group, ¥: Pearson's chi-squared test

## Discussion

We conducted a prospective double-blind, randomized controlled clinical trial involving patients, proposed for a scheduled surgery under general anesthesia with orotracheal intubation to compare the effect of two techniques: intravenous and intracuff lidocaine versus placebo in prevention of postoperative sore throat. We found that both intravenous and intracuff lidocaine improved the control of POST compared to placebo. They decreased the incidence of cough, dysphonia, dysphagia, nausea, and vomiting after surgery. However, they did not decrease the incidence of coughing during intubation. Postoperative sore throat is thought to be the main consequence of lesions induced by ischemia of the laryngotracheal mucosa. Stauffer [10] studied post-mortem tracheal lesions in 41 previously intubated patients. Edema and mucosal inflammation were the most common lesions (88% of patients), followed by ulcers (20%) and submucosal hemorrhages (14%).

Most of these lesions were located in the area of contact with the cuff of the tracheal tube. This is because the intubation tube cuff exerts pressure on the membrane of the vocal cords. A review of the literature and meta-analysis of Hockey CA [11] found that objective measurement-guided cuff pressure could prevent adverse effects of orotracheal intubation, including POST (OR 0.73; p <0.03), dysphonia, tracheal lesions, and microinhalation. In a meta-analysis of 3049 patients [12], lidocaine to inflate the tracheal tube cuff and intravenous lidocaine were effective in preventing POST. Lidocaine is believed to prevent stimulation of c fibers that would be excited by laryngoscopy and mobilization of the intubation tube. It is believed to prevent secondary neuroplasticity [13-15]. It would inhibit the secretion of tachykinin-like neuropeptides causing bronchoconstriction, cough, and sore throat [16]. A review of the literature and meta-analysis of Yang SS [6] found that intravenous lidocaine compared to placebo or without treatment resulted in a significant reduction in POST at the first hour (RR 0.46; 95% CI: 0, 32 - 0.67). It is difficult to determine the best time for IV administration of lidocaine. The subgroup analysis did not reveal any difference in the incidence of POST at the first hour with different dosages (p = 0.43) or the time of lidocaine administration (p = 0.91).

**Primary outcome:** we compared IV lidocaine administration with topical action after inflation of the ETT cuff with alkalinized lidocaine. We found that compared to placebo, intracuff alkalinized lidocaine significantly reduced VAS of the sore throat at the 6^th^ hour and the 24^th^ hour. However, Intravenous lidocaine reduced sore throat VAS only at the 24^th^ hour. Sconzo *et al*. [17] demonstrated a dose-dependent diffusion of lidocaine through the semi-permeable membrane of the ETT cuff. In fact, the cuff will serve as a reservoir, allowing the continuous administration of lidocaine in contact with the tracheal mucosa throughout the surgical procedure. Dollo *et al*. [7] carried out in vitro and in vivo studies on the lidocaine diffusion, whether in its acidic form marketed L-HCL or after alkalinization and neutralization of the pH. In the in vitro study, they proved that alkalinization makes it possible to reduce the dose of lidocaine needed to have the same diffusion profile, going from doses between 200 and 400 mg to doses between 20 and 40 mg, without waiting for a delay of 90 minutes of prefilling to saturate the polyvinyl chloride (PVC) membrane and thus have an immediate diffusion. A meta-analysis by Lam [5] with 1566 patients looked at the inflation of the ETT cuff with lidocaine and assessed the effect of this strategy on the phenomena of emergence. Eleven studies, with a total of 744 patients, focused on first hour POST, including 9 studies using alkalinized lidocaine. Among these 9 studies, 8 compared a control group using salines. The results showed that alkalinized lidocaine offered protection against POST (RR 0.33; 95% CI: 0.22-0,5). Alkalinized lidocaine to inflate the cuff was also able to prevent prolonged sedation and the delay in the emergence of anesthesia that may be seen with intravenous use of lidocaine [18]. It made it possible to have a better or comparable analgesic effect with lower plasma levels compared to intravenous lidocaine limiting any risk of systemic toxicity.

**Secondary outcomes:** in our trial, we found that there was a reduction in the incidence of coughing at intubation without being statistically significant: S: 13%, L: 17%, and T: 33% (p=0,126). This could be due to the lack of sedation monitoring and the possibility that orotracheal intubation was not performed at an adequate level of sedation. However, at the emergence of anesthesia, there was a statistically significant decrease in the incidence of cough in favor of intracuff alkalinized lidocaine and intravenous lidocaine with superiority to the first: S: 43%, L: 17%, and T: 73%. For postoperative cough at the sixth and the 24^th^ postoperative hours, the two techniques significantly reduced the incidence of these complications compared to the control group. Both techniques had similar efficacity. The cough in the perioperative period is generally transient. At the emergence of anesthesia, the cough would protect against bronchial inhalation but it would be responsible for perioperative morbidities [2] such as cardiac arrhythmias, hypertensive peaks, cardio and neurovascular complications, nausea, and vomiting. It is also responsible for complications that can affect the airways, such as iatrogenic bronchial ruptures during coughing. Coughing could increase intracranial, intra-abdominal, and/or intraocular pressure. Lidocaine is believed to be the most widely used substance in current practice for the prevention of POST and cough, and its efficacy was evaluated in a Cochrane review in 2015 [5,19]. Lidocaine is believed to reduce coughing by suppressing stimulation of sensory C fibers in the airways [14] and selectively depresses pain transmission to the spinal cord [13]. Clivio *et al*. [20] published a meta-analysis with 25 randomized clinical trials and a total of 3507 patients.

He concluded that intravenous lidocaine administration would reduce the incidence of cough during intubation as well as extubation and opioids induced cough. Four trials, involving 551 adults and 3 trials involving 185 children, investigated the effect of a single bolus of intravenous lidocaine on cough during intubation without curarization. According to Yang's meta-analysis [6] listing 13 clinical trials with 931 patients, the use of intravenous lidocaine resulted in a significant reduction in cough after extubation [RR: 0.64; 95% CI: 0, 48-0.86). Furthermore, the authors concluded that intravenous lidocaine is effective in preventing coughing during extubation. However, the PROSPERO meta-analysis and systematic review [5] concluded that the use of lidocaine in its L-HCL or alkalinized form to inflate the ETT cuff helped prevent POST and other orotracheal intubation morbidities. There was a decrease in the incidence of caught in intracuff group compared to the control group. These results were confirmed by the meta-analysis of Li H [12] the use of lidocaine to inflate the SIOT balloon decreased post-intubation cough and hoarseness of the voice. Peng *et al*. [2] found the same result (RR 0.45; 95% CI: 0.31 - 0.65; p <0.01).

We concluded the effectiveness of the use of lidocaine to prevent cough within 24 hours after surgery without difference between the two techniques. Inflating the ETT cuff with alkalinized lidocaine is better than the single bolus on induction to prevent coughing in extubation. There was no difference between these two to prevent cough during the intubation procedure. In our study, the incidence of dysphonia and dysphagia in the 6^th^ and 24^th^ hours was significantly reduced when we used alkalinized lidocaine to inflate the ETT cuff or a single bolus of lidocaine of 1,5 mg/kg during induction. There was no difference between the two techniques. The meta-analysis and review of the literature by Peng *et al*. [2] showed the effectiveness of alkalinized lidocaine in reducing the incidence of hoarseness (RR 0.44; 95% CI: 0.34 - 0.57; p <0.01) as well as dysphonia (RR 0.16; 95% CI: 0.06 - 0.46; p <0.01). Navarro *et al*. [21] found that this technique was ineffective for the smoker population. Unlike our trial, Hui Li *et al*. [12] found no effect of intravenous lidocaine on the incidence of hoarseness. Our study is a prospective double blinded clinical trial but it had few limitations. In Fact, we were unable to objectively monitor the pressure of the ETT cuff due to the lack of adequate manometers in our center that can measure the pressure of a liquid-filled cuff. The objective cuff pressure monitoring criteria were not an influencing factor in the sensitivity analysis for the meta-analysis by Lam *et al*. [5]. We were unable to objectively monitor the level of sedation due to the absence of a bispectral index (BIS), which could explain the difference in our results compared to the literature on cough during endotracheal intubation.

## Conclusion

Intravenous and alkalinized intracuff lidocaine were effective techniques to prevent postoperative sore throat and laryngeal morbidities in patients operated under general anesthesia with orotracheal intubation.

### What is known about this topic

- *Postoperative sore throat (POST) is a common complication after general anesthesia with endotracheal intubation;*
- *the use of local anesthetic solutions to inflate the endotracheal tube compared to placebo reduces postoperative sore throat;*
- *intravenous lidocaine compared to placebo significantly reduces postoperative sore throat*.

### What this study adds

- *Intravenous lidocaine reduces postoperative sore throat only at the 24^th^ postoperative hour*
- *Alkalinized lidocaine decreases postoperative sore throat at the 6^th^ and the 24^th^ postoperative hour with the lowest pain score compared to intravenous lidocaine;*
- *Both lidocaine techniques (intracuff and intravenous) reduce cough on emergence of anesthesia with a superiority of intracuff alkalinized lidocaine; both lidocaine techniques (intracuff and intravenous) decrease the incidence of cough, dysphonia, dysphagia and nausea vomiting in the first 24 postoperative hours compared to a placebo*.
